# Dynamic Impedance Analysis of Intestinal Anastomosis during High-Frequency Electric Field Welding Process

**DOI:** 10.3390/s22114101

**Published:** 2022-05-28

**Authors:** Li Yin, Caihui Zhu, Jianzhi Xu, Hui Zhao, Jian Qiu, Hao Wang, Kefu Liu

**Affiliations:** 1 School of Information Science and Technology, Fudan University, 220 Handan Road, Shanghai 200433, China; 19210720016@fudan.edu.cn (L.Y.); 20110720029@fudan.edu.cn (C.Z.); 20210720066@fudan.edu.cn (J.X.); hui_zhao@fudan.edu.cn (H.Z.); jqiu@fudan.edu.cn (J.Q.); 2 Academy for Engineering & Technology, Fudan University, 220 Handan Road, Shanghai 200433, China; 19210860056@fudan.edu.cn

**Keywords:** electrosurgery, high-frequency electric field welding, dynamic impedance, welding process, bursting pressure, compression pressure

## Abstract

The success rate of the electrosurgical high-frequency electric field welding technique lies in reasonable control of the welding time. However, the final impedance value used to control the welding time varies due to differences in tissue size and the welding method during the welding process. This study aims to introduce a new reference indicator not limited by impedance size from dynamic impedance to achieve an adequate weld strength with minimal thermal damage, providing feedback on the tissue welding effect in medical power supplies. End-to-end anastomosis experiments were conducted with porcine small intestine tissue under seven levels of compression pressure. The dynamic impedance changes were analyzed, combined with compression pressure, temperature, moisture, and collagen during welding. The welding process was divided into three stages according to the dynamic impedance, with impedance decreasing in Period Ⅰ and impedance increasing in Period Ⅲ. Period Ⅲ was the key to high-strength connections due to water evaporation and collagen reorganization. The dynamic impedance ratio is defined as the final impedance divided by the minimum impedance, and successful welding would be predicted when detecting the dynamic impedance ratio over 4 (*n* = 70, *p* < 0.001). Dynamic impedance monitoring can be used as a macroscopic real-time prediction of the anastomosis effect.

## 1. Introduction

Colorectal cancer ranks fourth in incidence and third in mortality worldwide; almost 555,477 new cases and 286,162 deaths occurred in China in 2020 [1]. Compared with needle sutures and staples for re-suturing in colorectal cancer resection, high-frequency electric field welding (H.F.E.W.) technology can improve the operative success rate and reduce surgical complications without additional material left [2,3]. As an energy-based electrosurgical suturing technique, H.F.E.W. rejoins tissues by pressure and electricity to make the collagen cross-linking restore continuity quickly [4,5,6]. LigaSure (Valleylab, Covidien) can reduce the surgical suture time to 5 s to fuse the colons under a radiofrequency power of 160 W [7]. Furthermore, Han et al. [8] used HFEW-300 PATONMED to complete the fusion, showing the potential to drastically simplify the surgical procedure and reduce the dependence on superior surgical skills.

However, an overdose of H.F.E.W. energy can cause excessive thermal damage, while an underdose cannot achieve sufficient weld strength. Therefore, it is necessary to determine when to stop energy output during the welding process by real-time parameters. Thermal damage can be visualized through microscopic level pathological sections [7]. Bursting pressure and axial tensile force are commonly used to evaluate the sealing quality [9,10,11,12,13]. However, these measurements are destructive to the anastomosis and are not suitable for use in actual surgery.

Dynamic impedance monitoring is a non-invasive, real-time parameter in the H.F.E.W. process. The existing electrosurgical suturing device controls the energy output by detecting the final impedance value [14,15,16]. Once the tissue coagulates and dries, the impedance rises to a critical level, and the current is then removed [17]. Tu et al. [18] set the final impedance to 50 Ω to achieve the maximum bursting pressures in porcine bowel end-to-end anastomosis. However, Wang et al. [19] proposed that a *R*_e_ range of 61.0~86.2 Ω was the optimum resistance with the highest connecting quality in automatic small bowel fusion. The final impedance inconsistency creates difficulties in selecting parameters for electrosurgical instruments. Even with the same tissue being welded by the same welding method, differences in isolation time, thickness, volume, water content, and measurement methods can lead to variations in impedance magnitudes [20,21]. So, the automatic control of the welding time using the final impedance does not apply to each welding process according to the individual differences.

Unlike a fixed value or range of final impedances, the impedance change rate is not limited by the impedance magnitude of the tissue. Li et al. [22] proposed a method based on the maximum impedance rate of change monitoring for vascular closure to terminate the sealing process. However, the impedance change rate measured in real-time is subject to fluctuating errors. Especially for tissues rich in water and fat (such as the intestine), the maximum impedance rate occurs with moisture and oil precipitation rather than well welding. Therefore, the calculation complexity, the misjudgment possibility due to fluctuation errors, and the individualization of the tissue limit the maximum-impedance-rate-of-change monitoring method.

Furthermore, dynamic impedance monitoring, both in terms of the final value and the change rate, lacks parameter measurements from different angles to explain the impedance changes during the H.F.E.W. process. To understand the welding process, we measured the variation of compressive force, surface temperature, moisture content, and collagen fibers with welding time. These parameters were used to explain the mechanism corresponding to the change in impedance during the welding process, thus identifying the important impedance characteristics for successful welding.

In this study, we used the relative change in impedance during welding to predict weld strength. When predicting weld strength using relative impedance values, differences in absolute values due to tissue size can be ignored. The ratio of final and minimum impedance during the H.F.E.W. process was first introduced to determine the proper welding time. Even if the initial compression pressure varies, success can still be statistically assured using dynamic impedance ratio monitoring. The feedback method of dynamic impedance ratio can solve the limitations of empirically judging the anastomosis effect, which is beneficial for realizing the adaptive medical power supply design and enhancing safety in the future.

## 2. Materials and Methods

In end-to-end inversion (i.e., serosa–serosa) anastomosis of the isolated porcine small intestine, the experiments contained H.F.E.W. experiments, impedance and other time-varying parameter measurements, and evaluation of the sealing quality.

### 2.1. Experimental Setup for H.F.E.W.

#### 2.1.1. Tissue Preparation

Fresh porcine small intestines were harvested from a slaughterhouse and transported to the laboratory stored in 0.9% isotonic saline at 0~4 °C. The mesentery was removed from the intestinal serosa before welding, and the interior contents were cleaned through washouts. The small intestines were then cut into roughly 100 mm long segments and soaked in saline. Each pair of small intestine segments in this study were anastomosed together within 24 h of isolation.

#### 2.1.2. H.F.E.W. Process

**Figure 1:**
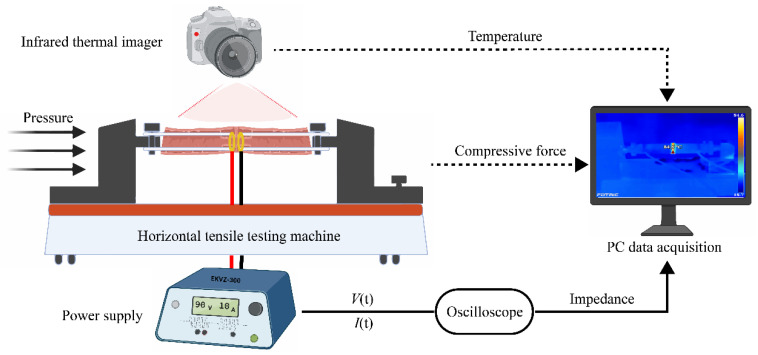
Tissue welding system with real-time monitoring impedance, pressure, and temperature. High-frequency electric field welding technology uses electricity and pressure to make two separated tissues quickly restore continuity. Two 100 mm long segments of small intestine tissue are turned inward over the ring electrodes, allowing the serosa–serosa anastomosis. The power supply delivers electrical energy to the welding area through the ring electrodes. The voltage and current are recorded by an oscilloscope and used to calculate dynamic impedance. The horizontal tensile testing machine applies pressure to the welding area through the column carrier and records the compressive force changes during the welding process. The infrared thermal imaging camera can monitor the temperature distribution on the weld surface during the welding process.

The real-time measurement system of intestinal tissue welding is shown in Figure 1. The power supply (EKVZ-300, PATONMED, Kyiv, Ukraine) was set to approximately 110 W to provide energy for tissue welding. The output voltage *V*(t) was measured by a differential probe (RP2015D, Rigol, Beijing, China) connected to a PC oscilloscope (6403D, PicoScope, Hardwick, UK), and the welding current *I*(t) by a current monitor (4100, Pearson, CA, USA). The dynamic impedance can be calculated by real-time voltage and current [19]. Temperature and pressure measurements that do not cause anastomosis rupture can be carried out simultaneously with welding while monitoring impedance during the welding process.

**Figure 2:**
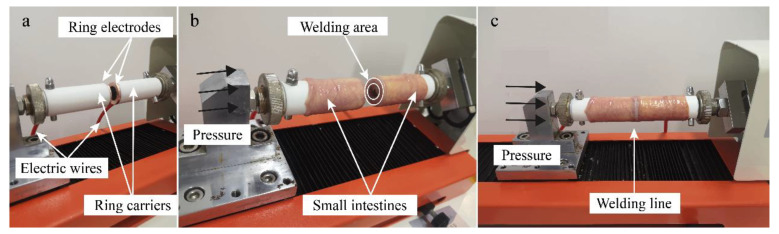
(**a**) Self-designed copper ring electrodes. The pair of self-designed copper ring electrodes has an inner diameter *d* of 13.31 mm and an outer diameter *D* of 23.09 mm. The electrodes are fixed onto the ring carriers of the horizontal tensile testing machine. (**b**) Small intestine welding area. Two sections of the small intestines are sleeved outside the ring carriers apart, and the welding area is the ring part of the small intestine covering the electrodes. (**c**) Welded intestines under pressure application. The serosal layers would be butted tightly under the set compression pressure, and the welding line would appear somewhat white after welding because of protein thermal denaturation.

The horizontal tensile testing machine (ZQ-980A, ZHIQU Precision Instruments, Dongguan, China) applies pressure to the end-to-end small intestines. In our study, the initial compression pressure (CP) for welding the small intestine had seven levels, 48 kPa, 78 kPa, 126 kPa, 205 kPa, 332 kPa, 536 kPa, and 868 kPa. The ratio between adjacent levels of CP was 0.618, making it easier to find the optimum CP range as quickly as possible. Figure 2a shows a pair of self-designed copper ring electrodes with an inner diameter *d* of 13.31 mm and an outer diameter *D* of 23.09 mm. So, the cross-sectional area *S* is

(1) $$S=\frac{1}{4}\pi \left(D^{2}{\;-\;d}^{2}\right)=279{.60\;\mathrm{mm}}^{2}$$

The electrodes were fixed onto the ring carriers of the horizontal tensile testing machine. Two sections of the small intestines were sleeved outside the ring carriers apart in Figure 2b, and the ring part of the small intestine covering the electrodes was the welding area. The serosal layers were butted tightly under the set CP, and the welding line showed slightly white after welding because of protein thermal denaturation in Figure 2c.

### 2.2. Impedance and Other Time-Varying Parameter Measurements

#### 2.2.1. Impedance Measurement

**Figure 3:**
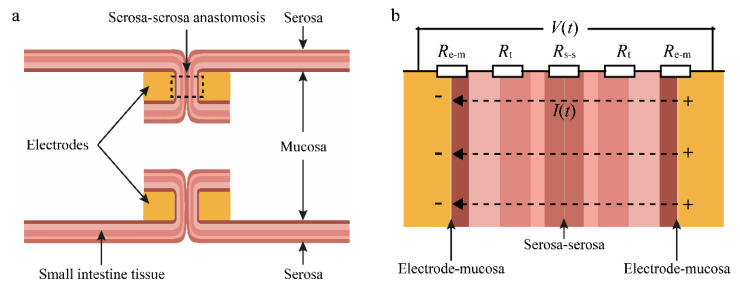
(**a**) Schematic diagram of the axial section in the welding area. The small intestinal mucosa comes into contact with the electrode, and the two serosa layers come into contact. (**b**) Impedance distribution between electrodes. The impedance value measured between the electrodes is the total of twice the electrode’s contact impedance to the mucosal layer *R*_e−m_, the contact impedance of double serosa layers *R*_s−s_, and twice the impedance of the small intestinal tissue itself *R*_t_. Changes in electrode spacing and small intestine thickness influence contact impedance, whereas changes in temperature, moisture, and collagen influence tissue impedance.

The impedance calculated by real-time voltage and current was measured between the two electrodes. Figure 3a depicts an axial schematic of the end-to-end anastomosis of small intestinal tissue. The mucosa of the small intestine was in contact with the electrode, and the two serosa layers were in contact in the welding area. The impedance value measured between the electrodes was the sum of twice the contact impedance of the electrode to the mucosal layer *R*_e−m_, the contact impedance of double serosa layers *R*_s−s_, and twice the impedance of the small intestinal tissue itself *R*_t_ in Figure 3b. The contact impedance was influenced by changes in electrode spacing and small intestine thickness, while the tissue impedance was related to changes in temperature, moisture, and collagen.

#### 2.2.2. Compressive Force Measurement

The contact impedance is related to the pressure between electrodes and the thickness of the tissue. The horizontal tensile testing machine can record the change in compressive force between electrodes during the welding process as well as the change in tissue thickness before and after welding at the same initial CP.

#### 2.2.3. Temperature and Moisture Content Measurement

**Figure 4:**
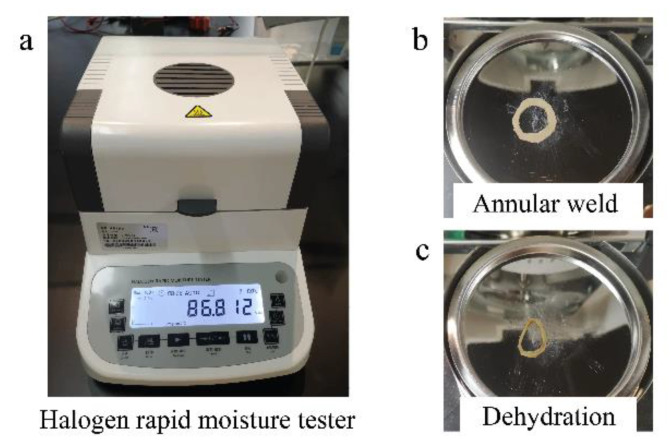
(**a**) Measurement of moisture content by the halogen rapid moisture tester. The decrease in moisture content causes a rapid increase in tissue impedance. (**b**) Annular weld after welding. Only the annular weld area of the small intestine is kept for moisture determination after welding. (**c**) Weld of the small intestine after complete dehydration. The weld of the small intestine loses its luster and elasticity due to the loss of surface free water and internal bound water after 100 °C complete dehydration.

The tissue impedance is macroscopically related to temperature and moisture content. The infrared thermal imaging camera (227s, FOTRIC, Shanghai, China) can monitor the temperature distribution on the weld surface during the welding process. The moisture content can be measured in different welding phases by the halogen rapid moisture tester (JK-10K, JIEKESI, Taizhou, China) in Figure 4a. Figure 4b shows the annular weld area of the small intestine ready for moisture determination after welding. Due to the loss of surface free water and internal bound water after 100 °C complete dehydration, the weld of the small intestine lost its luster and elasticity in Figure 4c and was significantly smaller in volume than in Figure 4b.

#### 2.2.4. Histological Study

Small intestines welded in different welding phases needed to be fixed in formalin immediately, processed in paraffin wax, and cut into slices transversely. Collagen at the weld was stained by Sirius Red to observe the changes in the collagen structure during the H.F.E.W. process. The prepared sections were mounted on the stereomicroscope (SZ680, Optics) to be visualized and imaged.

### 2.3. Sealing Quality Measurement

**Figure 5:**
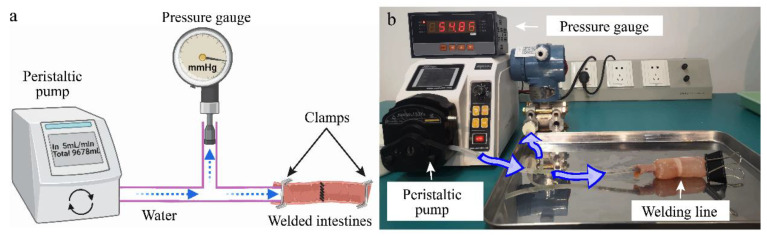
(**a**) Schematic diagram and (**b**) physical diagram of bursting pressure measurement device. One end of the welded small intestine is placed over the T-shaped tube, and the other end is completely sealed by a clamp. The peristaltic pump delivers water to the bowel cavity at a flow rate of 5 mL/min until the anastomosis leaks. The maximum water pressure was recorded as the bursting pressure by the pressure gauge. Bursting pressures below 15.4 mmHg (the highest peak pressure of the human ileum) are considered welding failures.

Figure 5a shows a schematic diagram of the postweld bursting pressure (BP) measurement. After discharging, the welded small intestine was removed, then with one end in the T-shaped tube and the other end completely sealed, water could pumped into the intestinal cavity at a rate of 5 mL/min by the peristaltic pump (BT-100CA 153Yx, JIHPUMP, Chongqing, China). The small intestine bulged due to water pressure in Figure 5b, and when the welding line leaked, the water pressure was recorded as the bursting pressure by the pressure gauge (YK-100, Shileke Technology, Xian, China). Welding failures were defined as BPs below 15.4 mmHg (the highest peak pressure of the human ileum). Analysis of variance (single-factor ANOVA, SPSS 26) was performed to examine the statistical difference in the small intestine BP with CP levels. Statistically significant was defined as *p*-value < 0.05.

## 3. Results

### 3.1. Dynamic Impedance

**Figure 6:**
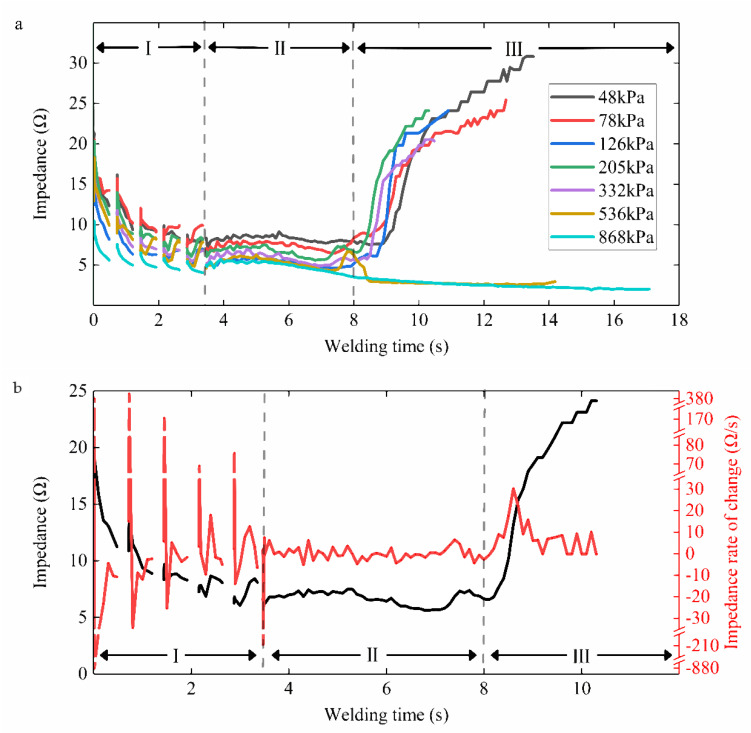
(**a**) Dynamic impedance under seven levels of initial compression pressure. The impedance curve is divided into three stages. The time of impedance reduction is Period Ⅰ, about 0−3.6 s. Period Ⅱ is the smooth period of impedance change, about 3.6−8 s. Impedance of Period Ⅲ shows two trends due to differences in initial compression pressure. The impedance would suddenly increase when the compression pressure is less than 332 kPa. There would be an inflection point due to water evaporation and collagen reorganization when Period Ⅲ is the key to high-strength connections. The impedance would continue to decrease under the pressure of 536 kPa and 868 kPa. (**b**) Impedance plot with the corresponding rate of change from small intestinal tissue of serosa–serosa anastomosis under 205 kPa. The rate of impedance change fluctuates very sharply in Period Ⅰ, far exceeding the rate of impedance rise in Period Ⅲ that follows. Relatively, the impedance itself is more stable and simpler to use as feedback.

The dynamic impedance curves over time under different CP levels are represented in Figure 6a. The impedance was calculated from real-time voltage and current, taking a value at least every 0.1 s to form an impedance curve. Each test was repeated at least ten times. However, differences in isolation time, thickness, volume, and water content may differ in the absolute value of tissue impedance. To study the impedance variation pattern that was not limited to the initial small intestine state, we focused on each welding process. Therefore, only a representative curve for each case is shown in Figure 6a. The impedance curve was divided into three stages. The time of impedance reduction was Period Ⅰ, about 0−3.6 s. Period Ⅱ was the smooth period of impedance change, about 3.6−8 s. The impedance of Period Ⅲ showed two trends under different CPs. The impedance would suddenly increase when the CP was less than 332 kPa, and we defined the moment between Period Ⅰ and Period Ⅱ as the impedance inflection point. However, the impedance kept decreasing under the pressure of 536 kPa and 868 kPa in Period Ⅲ, so there was no impedance inflection point in the impedance curve. In Figure 6b, the overall impedance under 205 kPa tended to initially reduce and then increased with the welding duration, representing the inflection point of mutation. The rate of change of impedance after the inflection point in Period Ⅲ could reach more than 30 Ω/s. However, in Period Ⅰ, the rate of impedance change fluctuated sharply, far exceeding the rate of impedance rise in Period Ⅲ. However, the impedance itself was relatively stable.

### 3.2. Compressive Force and Tissue Thickness

**Figure 7:**
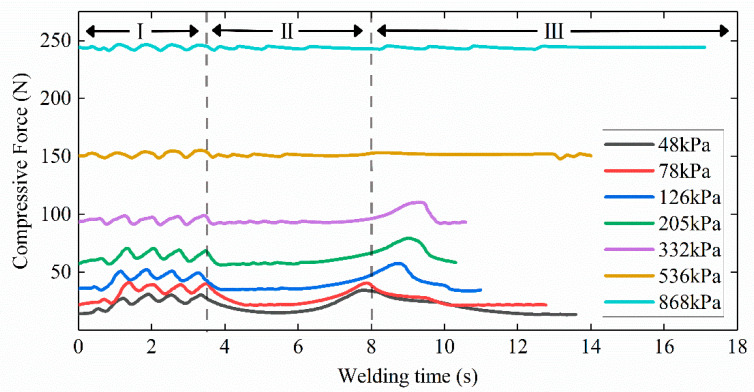
Compressive force between the electrodes during welding under seven levels of initial compression pressure. Pressure changes over time correspond to three stages of impedance change over time. Period Ⅰ is erratic, Period Ⅱ is steady, and Period Ⅲ is distinct due to differences in initial compression pressure. Compression force above the initial setting would cause the contact impedance between the electrode and the tissue to decrease.

The electrode spacing changed little throughout the welding process and was determined by the initial CP setting before welding. The pressure between the electrodes tended to return to the initial set pressure value during welding. When the intestine tissue between the electrodes swelled or curled, the compressive force change was time-varying during the welding process in Figure 7. The time variation in pressure coincided with three stages of impedance change with time. Period Ⅰ fluctuated frequently, Period Ⅱ was stable, and Period Ⅲ was different due to CP differences. The greater the initial CP, the smaller the pressure fluctuations generated by the thermal expansion of the small intestine, and the smaller the impact on the contact impedance between the electrodes and the tissue. However, when the pressure returned to the initial setting with the expansion ending in Period Ⅲ under CPs lower than 332 kPa, the small intestine tissue would shrink with the contact impedance increasing.

**Figure 8:**
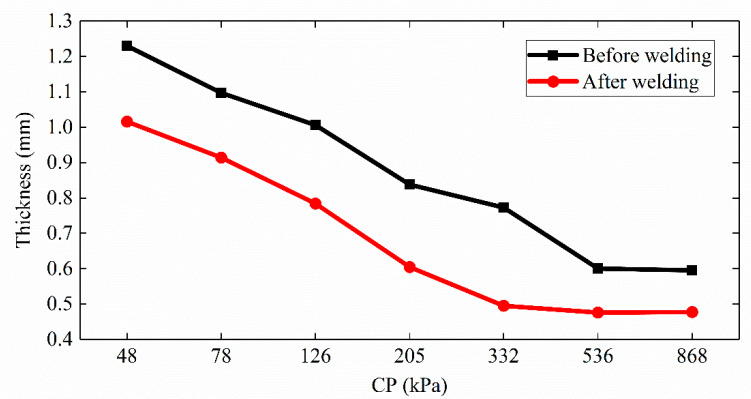
Thickness of double intestinal tissue before and after welding under seven levels of compression pressure (CP). When the CP is lower than 205 kPa, the tissue thickness after welding is smaller than the initial spacing between electrodes, increasing the contact impedance between the electrodes and the small intestine. Changes in tissue thickness have less effect on contact impedance above 332 kPa.

The thickness of the small intestine tissue (from the same pig) before and after welding changed in Figure 8 under different initial CPs. When the CP was lower than 205 kPa, the tissue thickness after welding was less than the initial spacing between electrodes. During welding, the reduced tissue thickness represented an increase in the contact impedance between the electrodes and the small intestine. The contact impedance itself was small under initial CPs above 332 kPa, and the pressure remained stable, so the reduction in tissue thickness had little effect on the contact impedance.

### 3.3. Surface Temperature and Moisture Content

**Figure 9:**
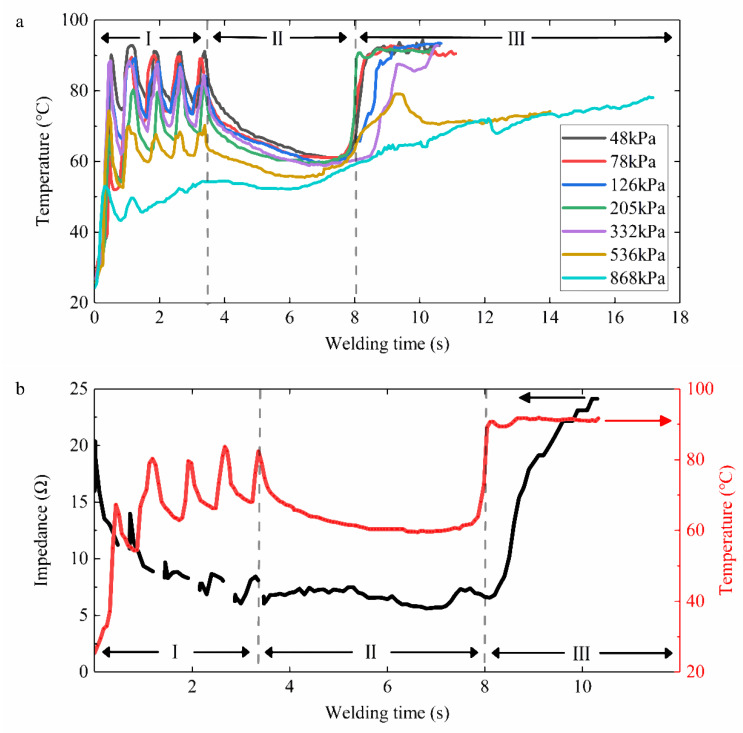
(**a**) Maximum surface temperature during welding under seven levels of compression pressure. Except that the temperature under 868 kPa rose slowly over time, the time variation in surface temperature corresponded to three stages of impedance change with time. The temperature in Period Ⅰ was fluctuating and increasing. The temperature in Period Ⅱ slowly fell to about 60 °C, and the temperature in Period Ⅲ rapidly rose to the maximum and remained there until the welding was completed. (**b**) Synchronous changes in impedance and temperature under 205 kPa. The temperature rise in Period Ⅰ caused the tissue impedance to decrease. The temperature rose sharply to the highest point at 8 s and remained high after that; the rapid increase in impedance in Period Ⅲ was due to tissue degeneration.

The surface temperature was highest at the center of the weld, where the two serosal layers of the small intestinal met. The surface temperature at the weld changing with time is recorded in Figure 9a. Except that the temperature under 868 kPa rose slowly over time, the time variation in surface temperature coincided with the three stages of impedance change with time. The temperature in Period Ⅰ fluctuated and rose. The temperature in Period Ⅱ slowly fell to about 60 °C, and the temperature in Period Ⅲ rapidly rose to the maximum and remained there until the welding was completed.

**Figure 10:**
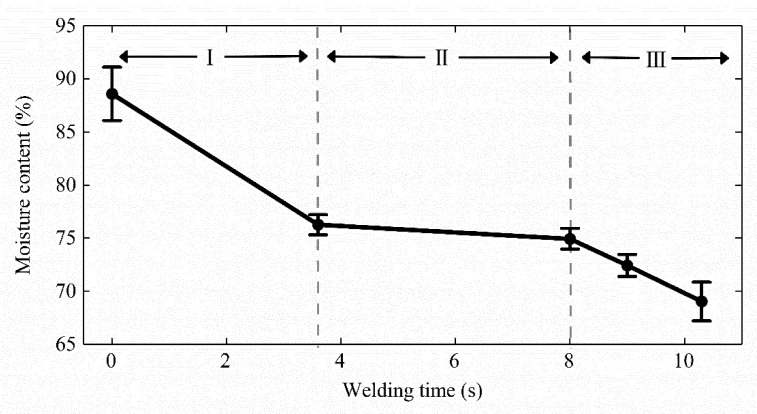
Moisture content during welding under the compression pressure of 205 kPa. The moisture evaporates intensely in Periods Ⅰ and Ⅲ but remained almost unchanged in Period Ⅱ. Water would be evaporated in the latter half of Period Ⅰ, increasing the tissue impedance. The impedance increased rapidly in Period Ⅲ, owing to the reduced moisture content weakening the conductivity.

Figure 9b shows the simultaneous change in surface temperature and impedance value under 205 kPa. The five pulse trains caused the temperature to rise in Period Ⅰ, and the impedance decreased. Period Ⅱ was a relatively stable stage, with a few fluctuations in temperature and impedance. The temperature rose sharply to the highest point at 8 s and stayed high after, and the impedance started to increase rapidly after the inflection point. Furthermore, the moisture content at the weld under 205 kPa decreased with the welding time in Figure 10. We can see that the moisture evaporated intensely in Periods Ⅰ and Ⅲ but was almost unchanged in Period Ⅱ.

### 3.4. Histological Observation and Collagen

**Figure 11:**
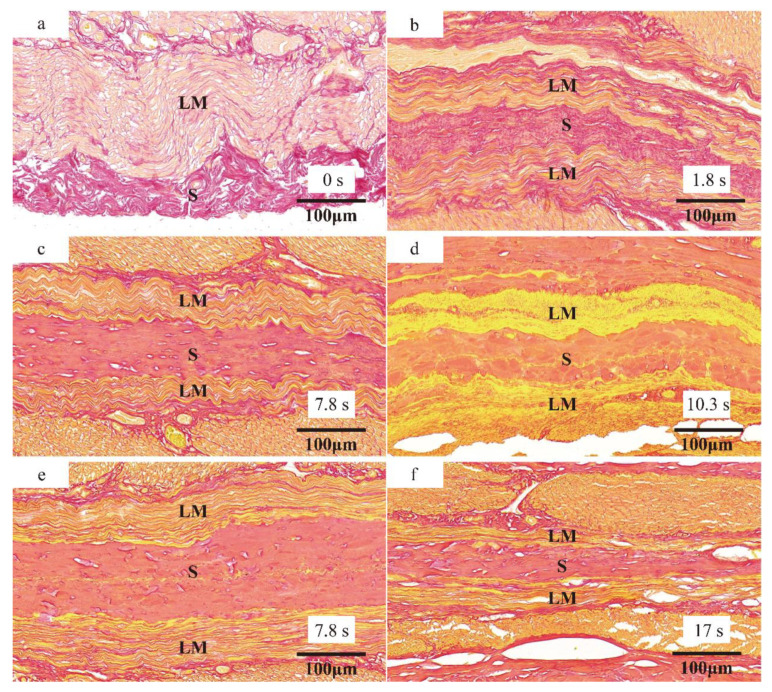
Longitudinal muscle (LM) and serosa (S) at the weld center: (**a**) blank control, 0 s; (**b**) 205 kPa, Period Ⅰ—1.8 s; (**c**) 205 kPa, Period Ⅱ—7.8 s; (**d**) 205 kPa, Period Ⅲ—9 s; (**e**) 868 kPa, Period Ⅱ—7.8 s and (**f**) 868 kPa, Period Ⅲ—13 s (Sirius red, magnification ×40.0). The serosal layer contains abundant connective tissue, with fibroblasts and collagen fibers essential for forming dense junctions. The collagen fibers of the serosal layer gradually merged from a free and disordered state before welding to compression and fusion in Periods Ⅰ and Ⅱ under 205 kPa, and even penetrated the longitudinal muscle layer in Period Ⅲ. Meanwhile, the collagen fibers of the LM layer changed from disordered to wavy to fused. The scattered collagen fibers re-formed dense connections in Period Ⅲ. However, the collagen fibers of the serosal layer and the LM layer did not interpenetrate at 868 kPa.

The serosal layer contains abundant connective tissues, in which fibroblasts and collagen fibers are critical to forming dense junctions. For serosa–serosa anastomosis, we focused on the change of longitudinal muscle (LM) and serosa (S) at the weld center over time, in Figure 11. The collagen fibers of the serosal layer under 205 kPa gradually changed from a free and disordered state before welding (Figure 11a) to a compressed state in Period Ⅰ (Figure 11b) and a fused state in Period Ⅱ (Figure 11c), and even penetrated the longitudinal muscle layer in Period Ⅲ (Figure 11d). Meanwhile, the collagen fibers of the LM layer changed from disordered to wavy to fused (Figure 11a–d) under 205 kPa but were smoother (Figure 11e,f) at a higher pressure of 868 kPa. Importantly, the collagen fibers of the serosal and the LM layers did not interpenetrate after welding in Figure 11f.

### 3.5. Dynamic Impedance Rate and Success Rate

We focused on the final impedance *R*_f_ and minimum impedance *R*_min_ for each welding process in this research. The final impedance *R*_f_ is the impedance value at the end of the weld, while the minimum impedance *R*_min_ occurs at the moment of inflection when the impedance suddenly increases during the impedance drop. To predict weld strength by impedance monitoring, a new index: dynamic impedance ratio *η*, is defined as

(2) $$\eta =\frac{R_{f}}{R_{\min}}$$

**Figure 12:**
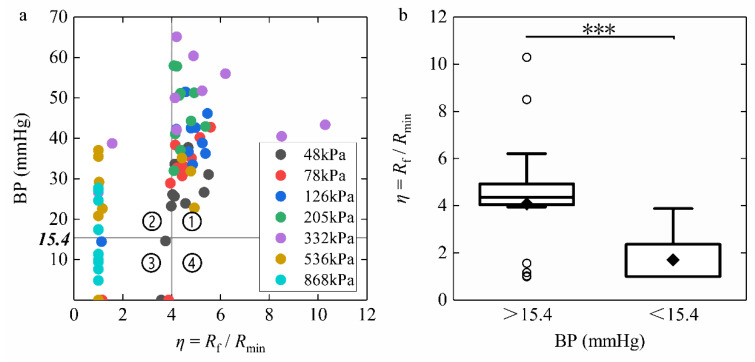
(**a**) Bursting pressure (BP) results affected by the ratio of final impedance to minimum impedance *η* with four quadrants. Quadrant ① shows that when *η* exceeds four, BP meets the success criteria despite differences in initial compression pressure. Although *η* is less than four, BP is successful in Quadrant ② over 332 kPa. The strength of the connection formed by prolonged heating is not stable, so there are also examples of unsuccessful cases in Quadrant ③. There is no data point in Quadrant ④, which indicates that BP failure would not occur when *η* is more than four. (**b**) BP results under *η* < 4 and *η* > 4. There is a significant difference in *η* with BP = 15.4 mmHg as the cut-off point (***: *p* < 0.001; ○: outliers; ⬥: means).

BP results are affected by the dynamic impedance *η* in Figure 12a. We divided Figure 12a into four quadrants based on the difference between BP success and *η* more or less than four. Quadrant ① shows that when *η* exceeds four, BP meets the success criteria despite CP heterogeneity. Although *η* is less than four, BP is successful in Quadrant ② under CP of over 332 kPa. However, the strength of the connection formed by prolonged heating is not stable, so there are also examples of unsuccessful cases in Quadrant ③. Quadrant ③ appears when the CP is too large or too small, *η* is less than four, and BP is unsuccessful. There is no data point in Quadrant ④, which indicates that BP failure would not occur when *η* is more than four under different CP levels. Figure 12b shows a significant difference in *η* with BP = 15.4 mmHg as the cut-off point (*p* < 0.001). Even without limiting the CP, welding success would be predicted for *η* higher than 4. If the optimal CP is determined, the overall level of BP for *η* over four will also increase.

**Figure 13:**
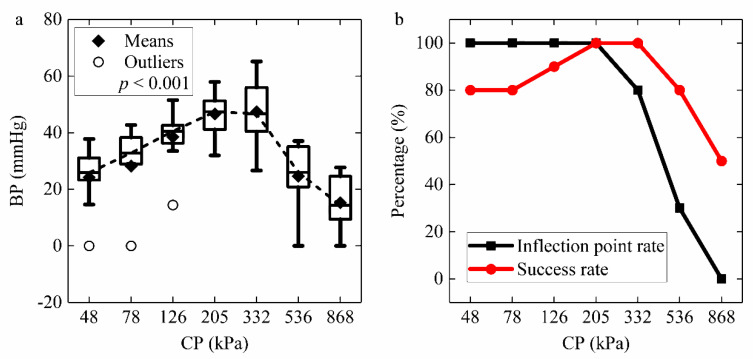
(**a**) Bursting pressure (BP) results and (**b**) inflection point rate and success rate under seven levels of compression pressure (CP). BPs under 205 kPa and 332 kPa are better than those under other CP levels. The optimal CP range for achieving the best anastomosis is 205 kPa to 332 kPa. The inflection point rate is defined as the number of inflection points per total number of experiments. The inflection point rate is higher than the success rate below 205 kPa but lower than over 205 kPa. Under the appropriate pressure of 205 kPa, the inflection point and success rates would increase to 100%. The dynamic impedance inflection point may be equivalent to successful welding and definite weld strength.

The distribution of BP with the CP is shown in Figure 13a. The highest BP was 47.46 ± 10.87 mmHg under 332 kPa, whereas the data under 205 kPa (46.62 ± 8.16 mmHg) was slightly lower but more stable. The BPs under 205 kPa and 332 kPa were better than those under other CP levels. It indicates that the optimal CP range is 205 kPa to 332 kPa to achieve the best anastomosis. Figure 13b compares the inflection point rate with the success rate under different CP levels. We define the number of inflection points per total number of experiments as the inflection point rate. If CP was too low, the inflection point rate was higher than the success rate. Whereas, when CP was too intense, the inflection point rate went down faster than the success rate. The inflection point rate and the success rate would both increase to 100% under the appropriate pressure of 205 kPa. It proves that the dynamic impedance inflection point may be equivalent to successful welding and definite connection strength.

## 4. Discussion

Dynamic impedance revealed the three stages of the tissue H.F.E.W. process in Figure 6a [17]. Firstly, the impedance dropped in Period Ⅰ. On the one hand, the increase in pressure caused the contact impedance to decrease (Figure 7), and on the other hand, due to the negative correlation between temperature and bio-impedance [23], the increase in temperature reduced the impedance of the small intestine (Figure 9a). The cells in the tissue might also perforate or rupture at this stage to enhance the fluidity and conductivity of cell fluid. Notably, a small amount of water would be evaporated in the latter half of Period Ⅰ, increasing the tissue impedance (Figure 10). Secondly, the pressure and temperature in Period Ⅱ remained stable, so the impedance fluctuated slightly. Still, the impedance reached the minimum when the temperature rose rapidly at about 8 s (Figure 9b). At the inflection point when the CP was less than 332 kPa, the temperature increase reduced the small intestinal tissue impedance (Figure 6a and Figure 9a). At the same time, the increase in pressure also reduced the contact impedance (Figure 6a and Figure 7) [24]. Finally, the impedance increased rapidly in Period Ⅲ, owing to the reduced moisture content weakening the conductivity (Figure 10) [25]. The inflection point was critical to achieving the perfect anastomosis effect. We also considered the scattered collagen fibers re-forming dense connections in Period Ⅲ (Figure 11d) [26]. In addition, as the thickness of the small intestine decrease after welding (Figure 8), the reduction in pressure to the initial set pressure value implied an increase in the contact impedance (Figure 7). However, when the CP was higher than 332 kPa, the contact impedance was unchanged because of little change in tissue thickness. So, the increase in temperature made the tissue impedance decrease all the time (Figure 9a).

The changes in impedance at various stages of the welding process were corroborated by pressure, temperature, moisture, and histological observation. Although pressure and temperature change with time in different phases, both pressure and temperature were relatively stable and insensitive in Period Ⅲ (Figure 7 and Figure 9a) before the welding was completed, so pressure and temperature cannot be used as an indication of when to stop the welding. The premise of bursting pressure, histological observation, and moisture detection would destroy the well-welded tissue and was therefore not considered. Accordingly, only using electrical impedance in the welding process was the most convenient feedback method.

The dynamic impedance can reflect the changes in the welding process in real-time, so we took the ratio of the final impedance to the minimum impedance *η* as a basis for judging the success of the welding. When *η* was four or over, the BP was higher than 15.4 mmHg (Figure 12a), which indicates a strong connection quality. The relative value of the impedance change may not be limited by the absolute value of the impedance of the tissue during welding. The anastomosis prediction adjusted according to each welding process was more reliable than stopping welding at a fixed value of 50 Ω [18]. Furthermore, dissimilar to Li’s research on dynamic impedance change rate [22], we believed that *η* was more intuitive and concise in judging the welding effect. The rate of impedance change fluctuated very sharply in Period Ⅰ (Figure 6b), far exceeding the rate of impedance rise in Period Ⅲ that followed. Therefore, using the maximum impedance rate of change as the basis for real-time monitoring for welding needs to be more careful. Relatively, the impedance itself is more stable and simpler to use as feedback.

Meanwhile, Figure 12b is further evidence that four times the minimum impedance can be used as a reliable prediction of successful BP. For vascular closure processes, *η* values are around 9 or even above 100 [22,25]. Although the coagulation strength may increase with a longer welding time, the overdose of the welding may aggravate the thermal damage and impede the future recovery of the tissue. Using the *η* range to determine the welding time can minimize thermal damage while ensuring successful welding. Even if the initial compression pressure changes, success can still be statistically assured using dynamic impedance ratio monitoring. If the optimal CP is determined, the overall level of BP for *η* over four will also increase.

This research concluded that the optimal CP range is 205 kPa to 332 kPa (Figure 13a). CP is a critical element in the tissue welding process and affects several outcomes, including the BP and the dynamic impedance. The initial CP setting that is too high or too low would cause a mismatch between the success rate and the inflection point rate (Figure 13b). Furthermore, for end-to-end anastomosis of the porcine bowel, Zhao [27] concluded the best CP was 277 kPa with BP of 44.6 ± 8.9 mmHg, which is slightly lower than our data (46.62 ± 8.16 mmHg at 205 kPa and 47.46 ± 10.87 mmHg at 332 kPa) in Figure 13a, but her sample size was only 22, so we conclude that the optimal pressure falls within our range. In addition, the best CP range of 0.10–0.25 MPa in mucosa-to-mucosa anastomosis differs from our serosa-to-serosa anastomosis [11]. Winter et al. [9] researched the pig colon rather than the small intestine, which is why they concluded that the optimal pressure was 1.125 N/mm^2^, namely 1125 kPa, much higher than other research outcomes. The optimal CP differs for various tissues with different connection methods. This paper, therefore, argues that the correct CP for serosa-to-serosa anastomosis of the porcine small intestine is between 205 and 332 kPa. Under this condition, we can predict a certain degree of tissue-welding strength via the dynamic impedance.

Monitoring the dynamic impedance ratio η to control the tissue welding time can achieve adequate weld strength on tissues with minimal thermal damage. The method can be used in automatic feedback devices for medical power supplies to enhance the success and safety of electrosurgery. Together with different surgical instruments, the method can also be useful for many surgery applications, such as small bowel closure or anastomosis [7,10,16,19], vascular closure [13,22], ablation of tumors [28], and chorioret-inal adhesion [29]. However, different welding strengths are required for different tissue types, so the corresponding dynamic impedance ratio values may need to be further explored.

## 5. Conclusions

The authors concluded that the ratio of the final impedance to the minimum impedance *η* was introduced to make the welding success rate up to 100% (*n* = 70, *p* < 0.001) under the optimal pressure range of 205 kPa to 332 kPa for the end-to-end anastomosis of the isolated pig small intestine with the bipolar high-frequency electric welding technology. Our data indicate that the bursting pressure would be successful when stopping welding with the final impedance four times the minimum impedance. In addition, we combined pressure, temperature, moisture, and histological observation to explain the impedance changes during welding. The dynamic impedance decreases in Period Ⅰ mainly because of the temperature rise, and the sudden increase of impedance in Period Ⅲ is a sign of high-strength connections between tissues due to water evaporation and tissue degeneration. Importantly, our results provide evidence for the conclusion that *η* rather than pressure, temperature, moisture, and histological observation can be served as power feedback to control welding time. It is expected that using *η* as a real-time impedance feedback mechanism for subsequent power supply design can realize the intelligent control of surgery to achieve optimal fusion. However, it is necessary to balance bursting pressure and thermal damage to study postoperative recovery. Accordingly, future research should be devoted to developing a medical power supply with impedance monitoring feedback to conduct in vivo experiments.

## Data Availability

The datasets generated during and/or analyzed during the current study are available from the corresponding author on reasonable request.
